# Identification of quantitative trait loci for phosphorus use efficiency traits in rice using a high density SNP map

**DOI:** 10.1186/s12863-014-0155-y

**Published:** 2014-12-31

**Authors:** Kai Wang, Kehui Cui, Guoling Liu, Weibo Xie, Huihui Yu, Junfeng Pan, Jianliang Huang, Lixiao Nie, Farooq Shah, Shaobing Peng

**Affiliations:** National Key Laboratory of Crop Genetic Improvement, Huazhong Agricultural University, Wuhan, 430070 Hubei China; MOA Key Laboratory of Crop Ecophysiology and Farming System in the Middle Reaches of the Yangtze River, College of Plant Science and Technology, Huazhong Agricultural University, Wuhan, 430070 Hubei China; Rice Research Institute, Guangdong Academy of Agricultural Science, Guangzhou, 510640 Guangdong China; Department of Agriculture, Anbar Campus of Abdul Wali Khan University, Mardan, Khyber Pakhtunkhwa Pakistan

**Keywords:** Genotype by environment interaction, Phosphorus use efficiency, Quantitative trait loci, Recombinant inbred lines, Rice, Single nucleotide polymorphism

## Abstract

**Background:**

Soil phosphorus (P) deficiency is one of the major limiting factors to crop production. The development of crop varieties with improved P use efficiency (PUE) is an important strategy for sustainable agriculture. The objectives of this research were to identify quantitative trait loci (QTLs) linked to PUE traits using a high-density single nucleotide polymorphism (SNP) map and to estimate the epistatic interactions and environmental effects in rice (*Oryza sativa* L.).

**Results:**

We conducted a two-year field experiment under low and normal P conditions using a recombinant inbred population of rice derived from Zhenshan 97 and Minghui 63 (*indica*). We investigated three yield traits, biomass (BIOM), harvest index (HI), and grain yield (Yield), and eight PUE traits: total P uptake (PUP), P harvest index (PHI), grain P use efficiency (gPUE) based on P accumulation in grains, straw P use efficiency (strPUE) based on P accumulation in straw, P use efficiency for biomass (PUEb) and for grain yield (PUEg) based on P accumulation in the whole plant, P translocation (PT), and P translocation efficiency (PTE). Of the 36 QTLs and 24 epistatic interactions identified, 26 QTLs and 12 interactions were detected for PUE traits. The environment affected seven QTLs and three epistatic interactions. Four QTLs (*qPHI1* and *qPHI2* for PHI, *qPUEg2* for PUEg, and *qPTE8* for PTE) with strong effects were environmentally independent. By comparing our results with similar QTLs in previous studies, three QTLs for PUE traits (*qPUP1* and *qPUP10* for PUP, and *qPHI6* for PHI) were found across various genetic backgrounds. Seven regions were shared by QTLs for yield and PUE traits.

**Conclusion:**

Most QTLs linked to PUE traits were different from those linked to yield traits, suggesting different genetic controls underlying these two traits. Those chromosomal regions with large effects that are not affected by different environments are promising for improving P use efficiency. The seven regions shared by QTLs linked to yield and PUE traits imply the possibility of the simultaneous improvement of yield and PUE traits.

## Background

Phosphorus (P) is an important nutrient for crops. Low levels of available P in soils and P use efficiency (PUE) are becoming the two major constraints for crop production. The addition of P to soils increases crop production costs, exhausts non-renewable P resources, and causes environmental problems [1-3]. Therefore, it is desirable to develop cultivars with high PUE.

Phosphorus use efficiency is improved by increasing both P uptake and use efficiencies [3,4]. For crops, P deficiency may be attributed to low total P content or low available P in soils; with respect to the latter, soils contain considerable amounts of P, but a large proportion is soil bound or in the organic form, and thus cannot be utilized by plants [5]. Therefore, improving PUE can be approached by various strategies. For soils with low total P content, strategies include regular applications of small doses of P and the improvement of internal PUE. Under P deficiency, crops have the ability to obtain more P through increasing the activities of enzymes involved in P scavenging and recycling and by altering respiratory electron transport and other metabolic pathways [1,6]. For soils with high unavailable P content, the most common strategy is the increase in P uptake efficiency through the proliferation and extension of plant roots, including the selection of deep rooting, thick roots, and strong root penetration ability [5]. Most investigations have focused on increasing P acquisition abilities by improving features such as root traits, root exudates, mycorrhiza, and high-affinity P transporters [7-10].

Among several parameters often investigated for P acquisition ability, P uptake (PUP) is an integrative trait that directly reflects a plant’s ability to acquire P. For example, Wissuwa et al. [3] used PUP to estimate P uptake efficiency and identified a major quantitative trait locus (QTL) named *Pup1* on chromosome 12 of rice. Further studies have shown that Nipponbare near-isogenic lines carrying *Pup1* could increase PUP in a severely P-deficient field, relative to the recurrent parent Nipponbare [11]. The high yield of modern rice varieties is mainly attributed to the high harvest index and great PUE for grain yield, which are due to the plants’ ability to mobilize P from vegetative to reproductive tissues [12]. However, the genetic relationship between grain yield formation and the traits associated with P uptake, re-translocation, and partitioning in plants have rarely been reported in previous studies [13,14].

Genome mapping can be used to locate the QTLs linked to PUE traits, which are controlled by multiple genes and show the genetic characteristics of quantitative traits [8,15,16]. In recent decades, QTLs for PUE and tolerance to P deficiency have been identified in rice [3,17], wheat [18], maize [19,20], and soybean [21,22]. Those studies mainly used two approaches. First, they focused on indirect traits, such as relative growth, relative tiller, root traits, shoot dry weight, and relative yield. Second, they investigated trait performance under P deficient conditions [8,17,23,24]. However, traits directly related to PUE, such as PUE for grain yield (PUEg), P harvest index (PHI), and P translocation efficiency (PTE), which are based on P uptake, grain yield, and biomass production, have rarely been used for evaluating and mapping QTLs [4,18,19].

Although dozens of QTLs have been identified in a single growing season in plants experiencing P-starvation, few QTLs have been detected in different years at the same or different locations, or in different genetic populations. Hence, as most traits are profoundly influenced by many genes and show various genotypes due to environmental interactions, few QTLs can be used to improve PUE [25]. Those QTLs detected for PUE traits or tolerance to P deficiency may be specifically functional or only fully expressed in a given environment. Furthermore, most previous investigations were carried out under controlled conditions or based on small crop populations with small experimental field plots [3,18,26]. Bray [27] emphasized that controlled conditions can never fully mimic field scenarios because crop plants grown in the field may face multiple abiotic and biotic factors. Therefore, more investigations on QTLs linked to PUE traits should be performed in several different environments.

Most QTL identifications in previous studies were based on linkage maps using restriction fragment length polymorphism (RFLP) and simple sequence repeat (SSR) markers. In those maps, sparse markers in many regions made it impossible to obtain precise and complete information about the number and locations of the QTLs [28]. The QTLs for PUE traits and P-deficiency tolerance in the previous studies were often located over a large confidence interval in RFLP/SSR maps, which complicated identification when there were several minor QTLs closely linked in the interval. Recently, new markers, such as single feature polymorphism (SFP) [29] and single nucleotide polymorphism (SNP) [28,30,31], have been used for QTL identification. These methods have facilitated the construction of high-density genetic maps, and have allowed precise and effective detections of QTLs in soybean, rice, and maize.

In most pervious reports, PUP was often used for estimating the P acquisition efficiency from soils; however PUEg or PUE for total biomass (PUEb) at maturity are better indicators in terms of biomass or grain yield. To investigate the physiological mechanisms of PUE, Rose and Wissuwa [32] suggested that it is preferable to dissect PUE into plant components, such as grain PUE (gPUE) and shoot PUE (strPUE). In addition, there is also significant P remobilization from leaves and stems during grain development to the developing grains [13]. Moreover, PHI is considered a parameter for PUE [33]. These three parameters, i.e., PHI, P translocation from stems to grains (PT), and P translocation efficiency (PE), are used to reflect P translocation from stems to grains during grain filling, which is associated with grain yield. In this study, a recombinant inbred population derived from a cross between Zhenshan 97 and Minghui 63, along with a high-density SNP bin map from Yu et al. [28], were used to locate QTLs linked to yield and eight PUE traits under two P application rates. The main objectives of the present study were (1) to locate QTLs for PUE traits and (2) to investigate the genetic relationship between PUE traits and yield traits and their stability across environments.

## Methods

### Plant materials and field experiments

The recombinant inbred line (RIL) population used in the study was derived by single-seed descent from a cross between two elite rice lines of *indica* subspecies, Zhenshan 97 and Minghui 63, the parents of Shanyou 63, the most widely cultivated hybrid in China [28,34]. Lines with too short a growth duration and with very low grain yield were not used for the field experiments because it is less significant to estimate PUE traits based on low grain yield or biomass from the viewpoint of crop production. Additionally, our experiment investigated PUE traits in large plots under real conditions for rice production. Thus, a total of 113 lines, plus the two parents, were used for the field experiments in 2008 and 2009.

The field experiments were carried out in the farmers’ field in Dajin town, Wuxue city, Hubei province, China (29°51' N, 115°33' E) during the rice-growing season from May to October in 2008 and 2009. The soil type was gleyed paddy soil, and it exhibited the following properties in the top 25 cm: pH 5.20, 25.89 g kg^−1^ organic C, 1.57 g kg^−1^ total N, 5.35 mg kg^−1^ available Olsen-P, and 54.93 mg kg^−1^ exchangeable K.

The experiments were conducted following a randomized complete block design with three replicates. Each replicate contained two P application rates: low P (without P fertilizer) and normal P applications (with pure P of 40 kg ha^−1^, equal to 92 kg P_2_O_5_ ha^−1^). All the P was applied as basal fertilizer in the form of calcium superphosphate one day before transplanting. To support high grain yield, a total of 135 kg N ha^−1^ in the form of urea was applied three times: 54 (40%) kg ha^−1^ as basal fertilizer, 40.5 (30%) kg ha^−1^ 15 days after transplanting (DAT), and 40.5 (30%) kg ha^−1^ 25 DAT. Potassium (100 kg K ha^−1^ as potassium chloride) was applied two times: 50 kg ha^−1^ as basal fertilizer and 50 kg ha^−1^ 25 DAT. Zinc (5 kg Zn ha^−1^) was applied in the form of zinc sulfate heptahydrate as basal fertilizer. Under the low P application, the applications of the other fertilizers were the same as those under the normal P application. All the fertilizers were applied during an early growth stage.

In both years, seeds were sown in nursery plastic plates on 17 May and the seedlings were transplanted on 15 June. Each line was transplanted to plots with a spacing of 0.20 × 0.17 m and an area of 8.2 m^2^. Each plot included 14 rows with 16 hills per row and three 27-day-old seedlings per hill. To minimize seepage between the P applications, the main plots were separated with double bunds to prevent water flow, and all the bunds were covered with plastic film, extending to a depth of 20 cm below the soil surface. To avoid loss and movement of the fertilizers, the plots were not drained during the duration of the experiment. A flood-irrigation system was adopted, which followed high-yield agricultural practices according to the local rice production. Pests, diseases, birds, and weeds were intensively controlled.

### Sampling and measurements

At the heading of the plants from each line (plot), eight uniform plants were sampled (excluding the border plants). The plants were separated into leaves and stems (including culms, sheaths, and young panicles). At maturity, twelve uniform plants were harvested from the middle of each plot. All the panicles were collected and hand-threshed, and then all the grains were divided into filled and unfilled groups by submerging them in tap water. All the leaves, stems (culms and sheaths), rachis, and filled and unfilled grains were separately collected and oven-dried at 80°C until a constant weight was achieved. The grain yield (Yield, g m^−2^) was reported at a 14% moisture content basis. The harvest index (HI, %) was calculated as the ratio of grain dry matter to total aboveground biomass (BIOM, g m^−2^). The BIOM, Yield, and HI were considered as yield traits in the study.

### Measurements of P concentration

The oven-dried leaves, stems, and filled grains were separately grinded into powders and mixed thoroughly, and each powder was passed through a 1-mm sieve. Approximately 0.2 g of each sample powder was digested with sulfuric acid and hydrogen peroxide to determine the P concentration spectrophotometrically according to the molybdenum blue method [35], using a continuous-flow analyzer (FUTURA, Alliance Instrument, France). The P concentration was calculated based on dry weight.

### Definitions of PUE traits

**Figure 1:**
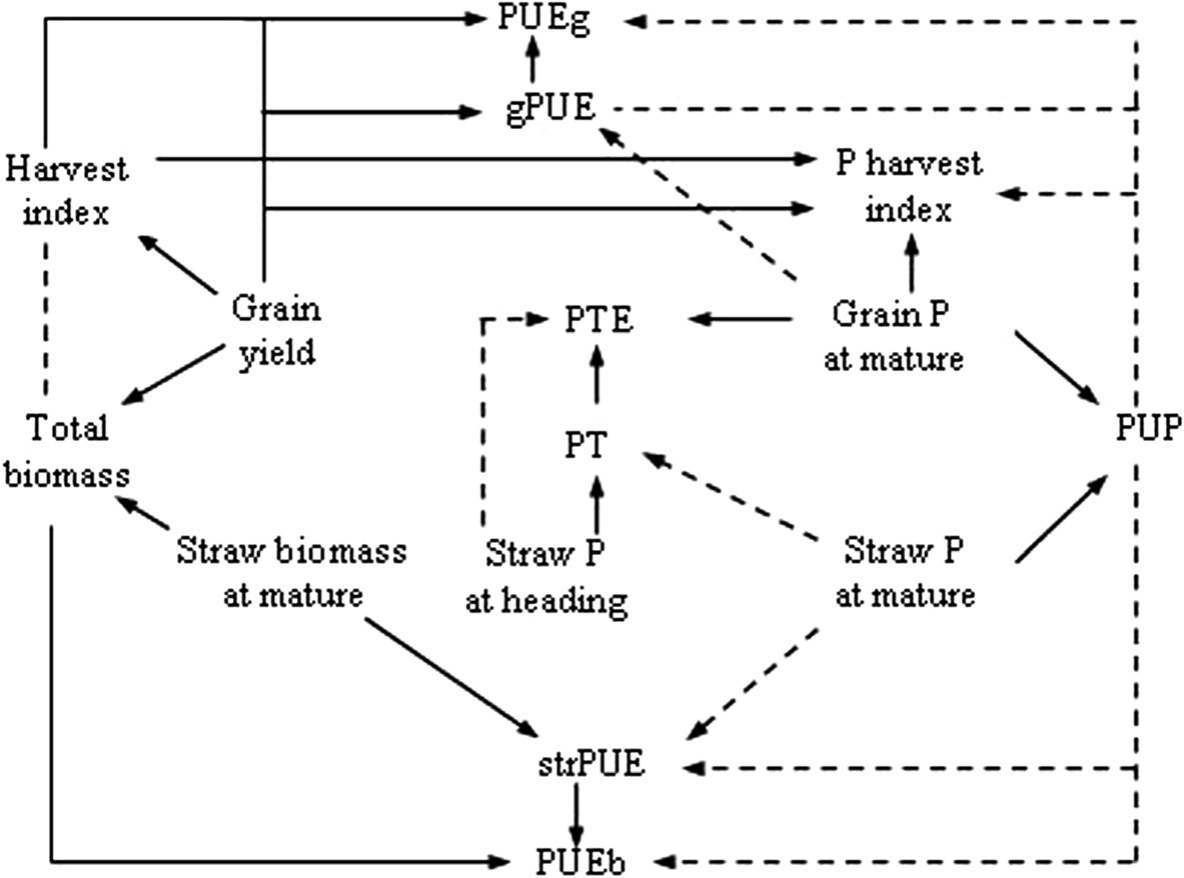
**The three yield traits and eight P use efficiency traits and their inter-relationships.** gPUE: P use efficiency for grain yield based on P accumulation in grains, PT: P translocation, PTE: P translocation efficiency, PUP: total aboveground P uptake, PUEb: P use efficiency for biomass accumulation, PUEg: P use efficiency for grain yield, strPUE: P use efficiency for straw dry weight based on P accumulation in straws.  represents a positive relationship between two traits,  represents a negative relationship,  represents no obvious relationship.

Eight PUE traits were calculated, as described by Dordas [13], Jones et al. [33], and Rose and Wissuwa [32]. The P accumulation of each plant part (including leaves, stems, and grains) is the product of the part’s dry weight and its corresponding P concentration. The P uptake at maturity (PUP, g m^−2^) is the sum of the accumulations in the various plant parts per m^2^. The P harvest index (PHI, %) is the ratio of the grain P accumulation to the total P accumulation of the aboveground parts at maturity. The grain PUE (gPUE, g g^−1^) is defined as the filled grain dry weight per g P in grains. The straw PUE (strPUE, g g^−1^) is defined as the straw dry weight per g P in straw at maturity. The PUE for biomass (PUEb, g g^−1^) is defined as the total aboveground biomass per g P accumulated in the whole plant at maturity. The PUE for grain yield (PUEg, g g^−1^) is defined as the grain yield per g P accumulated in the whole plant at maturity. The P translocation from stems to grains (PT, g m^−2^) is calculated as the leaf and stem P accumulations at heading minus those at maturity. The P translocation efficiency (PTE, %) is defined as the ratio of PT to P accumulation at heading. A network diagram for the investigated PUE traits are presented in Figure 1.

### Phenotype data analysis

The means over three replicates were used for all the statistical analyses, which were conducted using SAS 9.1 (North Carolina, USA). The broad-sense heritability (*h*_*B*_^2^, %) based on the RILs was estimated according to the following formula: *h*_*B*_^2^ = *σ*^2^_*g*_/(*σ*^2^_*g*_ + *σ*^2^_*ge*_/*e* + *σ*^2^_*e*_/*re*), where *r* is the number of replicates per year, *e* is number of environments (years), *σ*^*2*^_*g*_ is the genetic variance, *σ*^*2*^_*ge*_ is the variance of genotype due to environmental interactions, and *σ*^*2*^_*e*_ is the residual variance [36].

### Construction of genetic linkage map and QTL detection

A high-density bin map based on SNP, as described by Xie et al. [37] and Yu et al. [28], was constructed. The map consisted of 1619 recombination bins, covering all chromosomes without missing data and spanning 1625.5 cM in length, with an average interval of 1.0 cM between adjacent SNP markers. All the markers were used for the QTL mapping analysis in this study. Due to the large number of SNP markers, the markers not involved in candidate QTLs or epistatic interactions were removed from the genetic map figures.

As revealed by the analysis of variance, the different P applications (low and normal) and study years (2008 and 2009) had different effects on the traits. Thus, the four combinations of these factors (two years and two P applications) were considered as four environmental factors (e1 and e2 represented low P in 2008 and 2009; e3 and e4 represented normal P in 2008 and 2009). The mean of three replicates of each P application and each year were considered as the phenotypic score for each environment. All of the phenotype data were normally distributed and directly used for the QTL detection without any transformations. The QTL detection was performed with the QTLNetwork-2.0 software (Institute of bioinformatics, Zhejiang University, Hangzhou, China, Yang and Zhu 2005) based on a mixed linear model [38]. Composite interval analyses were conducted with a 10 cM window size and a 0.5 cM walking speed. One thousand permutations were performed for each trait to calculate a critical F value at *P* < 0.05. The Monte Carlo Markov Chain was applied to estimate the QTL effects. A QTL was declared if the phenotype was associated with a marker locus at *P* < 0.005. The QTL naming followed the procedure presented by McCouch et al. [39]. Additive × additive effects (aa), additive × environment interactions (ae), and additive × additive × environment interactions (aae) were separately estimated using the QTL location software.

Individual and multi-environment (e1, e2, e3, and e4) combined analyses were performed, and the locations and effects of the QTLs were compared between individual and combined analyses. The locations and effects of QTLs were reported, as well as the epistatic interactions detected by the combined analysis across the four environments. Additionally, we also clarified the individual environments for which similar QTLs for a given trait were identified in the same or neighboring region by individual environment analysis.

## Results

### Phenotypic variation

Under both low and normal P conditions in both two years, Minghui 63 had higher BIOM, Yield, PUP, PUEb, and PT, but lower HI, PHI, PUEg, and PTE than Zhenshan 97 (Table 1). With the exception of PUEg and PT for Zhenshan 97 under low P, the two parents had higher BIOM, HI, Yield, PUP, PHI, PUEg, PT, and PTE in 2008 than in 2009 under both P applications. Under low P, the PHI, gPUE, strPUE, PUEb, PUEg, and PTE of the two parents were higher than under normal P in both years. Minghui 63 had a higher PT under low P compared with under normal P; however, the opposite was true for Zhenshan 97.

**Table 1:** **Mean, range, and heritability for yield and P use efficiency traits of the population**

**Trait**	**Year**	**Parents**			**RILs**					
		**Minghui63**	**Zhenshan97**	**LSD**_**0.05**_	**Mean**	**Min**	**Max**	**Skew**	**Kurt**	***h***_***B***_^***2***^
**Low P**										
BIOM	2008	1585	819	378	1224	715	1678	0.15	1.08	90.9
(g m^−2^)	2009	1391	795	146	1211	789	1468	−0.74	0.71	
HI	2008	44.1	48.3	7.7	40.7	27.7	51.6	−0.16	0.00	67.5
(%)	2009	36.4	43.9	15.2	40.0	24.8	51.4	−0.51	0.12	
Yield	2008	813	464	255	580	335	1000	0.67	1.15	72.2
(g m^−2^)	2009	587	407	217	558	163	783	−0.59	1.20	
PUP	2008	3.83	2.13	0.93	3.17	2.05	4.94	0.46	1.98	85.0
(g m^−2^)	2009	3.02	1.83	1.25	2.75	1.89	3.45	−0.61	0.24	
PHI	2008	64.3	72.6	3.1	58.7	36.2	74.1	−0.37	−0.20	74.3
(%)	2009	56.5	63.0	25.1	57.4	18.6	73.7	−0.91	1.83	
gPUE	2008	285	260	108	270	221	351	0.84	1.16	54.9
(g g^−1^)	2009	304	311	109	309	255	404	0.55	1.09	
strPUE	2008	650	730	199	582	394	862	0.61	−0.30	77.4
(g g^−1^)	2009	743	695	575	666	463	1005	0.62	0.01	
PUEb	2008	415	388	124	390	330	490	0.69	0.29	75.7
(g g^−1^)	2009	472	447	188	447	367	573	0.68	0.75	
PUEg	2008	213	219	92	184	121	254	0.22	−0.25	67.6
(g g^−1^)	2009	204	230	157	207	55	295	−0.49	1.51	
PT	2008	1.94	0.98	0.57	1.49	0.68	2.18	0.21	0.18	70.2
(g m^−2^)	2009	1.63	1.16	0.39	1.29	0.43	2.06	−0.33	−0.21	
PTE	2008	61.0	76.5	10.1	61.0	25.4	80.2	−0.43	0.38	80.9
(%)	2009	58.5	76.1	26.3	58.3	16.4	79.0	−0.95	1.05	
**Normal P**										
BIOM	2008	1610	810	200	1269	801	1704	−0.49	1.00	86.9
	2009	1395	790	277	1232	808	1513	−0.78	0.97	
HI	2008	42.5	50.7	6.3	40.8	27.7	50.0	−0.24	−0.44	72.3
	2009	31.8	44.8	5.8	38.9	23.2	49.6	−0.54	−0.14	
Yield	2008	796	479	148	600	197	908	0.07	1.20	69.4
	2009	514	409	83	553	179	757	−0.57	0.76	
PUP	2008	4.30	2.56	0.76	3.49	2.24	4.73	0.01	0.94	83.8
	2009	3.78	2.22	0.73	3.29	2.29	4.21	−0.09	−0.28	
PHI	2008	59.1	67.8	4.8	54.8	35.0	70.4	−0.21	−0.52	78.2
	2009	42.7	56.7	4.0	50.0	14.4	68.0	−0.70	0.91	
gPUE	2008	271	239	89	273	217	343	0.38	0.52	76.1
	2009	274	284	65	292	236	332	0.20	−0.24	
strPUE	2008	530	484	106	492	304	757	0.52	0.31	81.2
	2009	438	457	70	474	326	664	0.45	−0.30	
PUEb	2008	377	317	81	364	272	469	0.23	1.75	80.4
	2009	368	359	61	377	300	465	0.27	0.01	
PUEg	2008	187	188	63	173	88	231	−0.10	0.09	73.5
	2009	136	187	45	170	45	246	−0.57	0.76	
PT	2008	1.68	1.55	1.23	1.55	0.79	2.48	0.09	−0.08	58.7
	2009	1.39	1.27	0.29	1.44	0.36	2.28	−0.68	0.81	
PTE	2008	50.0	75.8	23.6	56.5	29.8	77.3	−0.49	−0.28	80.9
	2009	41.2	66.7	13.1	51.6	13.4	76.0	−0.83	0.55	

BIOM: total aboveground biomass, HI: harvest index, gPUE: P use efficiency for grain yield based on P accumulation in grains, PHI: P harvest index, PT: P translocation, PTE: P translocation efficiency, PUP: total aboveground P uptake, PUEb: P use efficiency for biomass accumulation, PUEg: P use efficiency for grain yield, strPUE: P use efficiency for straw dry weight based on P accumulation in straw, *h*_*B*_^2^: broad heritability.

All the traits varied widely under both the low and normal P applications in both years (Table 1), and the transgressive variations were both positive and negative. The broad-sense heritabilities for all 11 traits varied widely, ranging from 54.9% for gPUE to 90.9% for BIOM under low P, and from 58.7% for PT to 86.9% for BIOM under normal P (Table 1). Generally, BIOM, PUP, and PTE had high heritabilities, whereas gPUE, PUEg, and PT showed low heritabilities. The 11 traits varied significantly with year, P level, and genotype (Table 2).

**Table 2:** **Analysis of variance for yield and P use efficiency traits**

**Source**	***df***	**F-value**										
		**BIOM**	**HI**	**Yield**	**PUP**	**PHI**	**gPUE**	**strPUE**	**PUEb**	**PUEg**	**PT**	**PTE**
Year (Y)	1	19.5**	153.7**	145.9**	272.0**	115.1**	373.8**	33.0**	282.4**	74.8**	84.0**	82.4**
P level (P)	1	36.9**	13.8**	7.2**	516.8**	395.6**	22.5**	605.7**	515.5**	405.5**	35.6**	177.1**
Genotype (G)	112	26.8**	56.0**	37.1**	12.1**	25.1**	5.2**	8.1**	5.9**	18.8**	7.8**	19.5**
Y × P	1	3.0	24.7**	21.3**	31.6**	38.5**	31.9**	78.4**	109.7**	116.8**	5.0*	8.5**
Y × G	112	2.2**	16.1**	10.2**	1.2	5.6**	1.4**	1.5**	1.0	5.1**	2.5**	2.9**
P × G	112	0.8	1.7**	1.4*	1.1	1.1	1.1	1.0	1.2	1.1	1.5**	1.1
Y × P × G	112	0.9	1.2	1.0	0.8	0.7	0.9	0.5	0.6	0.8	0.9	1.0

See Table 1 for abbreviations. * and **Indicate significance at *P* = 0.05 and *P* = 0.01, respectively.

### Correlation among various traits

For all traits, significant positive correlations were observed between low and normal P applications in 2008. Similar correlations were also found in 2009. These correlations ranged from 0.40 for gPUE to 0.90 for both Yield and HI in 2008, and from 0.64 for PUEb to 0.93 for HI in 2009 (Table 3). Significant positive correlations were also found between all trait values from 2008 to 2009 under both P applications.

**Table 3:** **Correlations among yield and P use efficiency traits**

**Trait**	**BIOM**	**HI**	**Yield**	**PUP**	**PHI**	**gPUE**	**strPUE**	**PUEb**	**PUEg**	**PT**	**PTE**
2008											
BIOM	0.89** ^**a**^	*0.14*	0.76**	0.77**	*0.14*	0.39**	0.24*	0.42**	0.31**	*0.03*	−0.32**
HI	0.10	0.90**	0.74**	*0.01*	0.85**	*0.15*	0.48**	0.19*	0.86**	0.41**	0.50**
Yield	0.76**	0.71**	0.90**	0.53**	0.63**	0.36**	0.46**	0.39**	0.76**	0.30**	*0.11*
PUP	0.78**	0.11	0.63**	0.74**	*−0.17*	*0.07*	−0.31**	−0.25**	*−0.13*	*−0.07*	−0.57**
PHI	0.12	0.83**	0.60**	−0.04	0.86**	*−0.09*	0.81**	0.45**	0.89**	0.39**	0.66**
gPUE	0.18	0.05	0.16	−0.15	−0.24**	0.40**	*0.07*	0.49**	0.37**	*0.03*	*−0.12*
strPUE	0.22*	0.42**	0.40**	−0.25**	0.78**	0.01	0.78**	0.82**	0.79**	0.26**	0.58**
PUEb	0.28**	0.01	0.18	−0.36**	0.25**	0.53**	0.73**	0.56**	0.65**	*0.12*	0.31**
PUEg	0.23*	0.82**	0.68**	−0.12	0.81**	0.36**	0.76**	0.57**	0.80**	0.38**	0.56**
PT	0.27**	0.24*	0.37**	0.21*	0.20*	0.11	0.16	0.10	0.26**	0.59**	0.75**
PTE	−0.29**	0.45**	0.10	−0.48**	0.62**	−0.03	0.59**	0.32**	0.56**	0.57**	0.82**
2009											
BIOM	0.89**	*−0.13*	0.51**	0.73**	*−0.08*	0.30**	*0.17*	0.36**	*0.04*	*0.00*	−0.32**
HI	−0.11	0.93**	0.78**	−0.33**	0.91**	*0.18*	0.53**	0.27**	0.91**	0.63**	0.73**
Yield	0.49**	0.81**	0.90**	*0.18*	0.73**	0.34**	0.55**	0.44**	0.81**	0.55**	0.44**
PUP	0.77**	−0.18	0.3**	0.78**	−0.42**	*−0.08*	−0.44**	−0.37**	−0.42**	−0.35**	−0.67**
PHI	−0.06	0.93**	0.76**	−0.25**	0.90**	*−0.02*	0.79**	0.47**	0.92**	0.68**	0.81**
gPUE	0.12	0.25**	0.30**	−0.28**	0.10	0.70**	*0.14*	0.52**	0.37**	*0.18*	*0.09*
strPUE	0.11	0.56**	0.54**	−0.38**	0.77**	0.28**	0.76**	0.85**	0.79**	0.60**	0.68**
PUEb	0.22*	0.11	0.21*	−0.44**	0.29**	0.61**	0.76**	0.64**	0.64**	0.47**	0.47**
PUEg	0.00	0.89**	0.78**	−0.35**	0.91**	0.50**	0.82**	0.54**	0.88**	0.70**	0.78**
PT	0.29**	0.59**	0.69**	0.15	0.56**	0.19*	0.37**	0.14	0.56**	0.67**	0.89**
PTE	−0.24**	0.80**	0.56**	−0.46**	0.83**	0.28**	0.65**	0.34**	0.83**	0.74**	0.84**
Between 2008 and 2009											
Low P	0.85**	0.52**	0.57**	0.76**	0.60**	0.38**	0.63**	0.61**	0.54**	0.55**	0.69**
Normal P	0.78**	0.61**	0.57**	0.73**	0.67**	0.63**	0.69**	0.68**	0.63**	0.42**	0.70**

See Table 1 for abbreviations.

The bold value in the diagonal indicates correlations between low and normal P values for an identical trait. The values below the diagonal are correlations under the low P application, and the values above the diagonal are correlations under the normal P application.

* and **Indicate significance at *P* = 0.05 and *P* = 0.01, respectively.

The main inter-relationships among the 11 investigated traits are presented Figure 1. Generally, three yield traits were significantly correlated with PUE traits (Table 3). There were similar correlations among the eight PUE traits under low and normal P applications in both years, separately. Under both the low and normal P applications, PUP was negatively correlated with strPUE, PUEb, and PTE in each year; however, PUP was negatively correlated with PHI and PUEg in 2009 only. Moreover, PHI was positively correlated with strPUE, PUEb, PUEg, PT, and PTE. Under the two P applications, gPUE was positively correlated with PUEb and PUEg in each year. Positive correlations between strPUE and PUEb and between PUEg and PTE under the two P applications in each year were found. Under the two P applications in each year, PUEb was positively correlated with PUEg and PTE, and PUEg was positively correlated with PT and PTE.

### QTL detection

Based on the multi-environment combined analysis, a total of 36 QTLs were detected for the 11 investigated traits (Table 4), 26 of which also detected in the similar regions by individual environment analysis. Compared with the QTL locations determined by individual environment analysis, seven of the 36 QTLs were simultaneously detected in both years, 14 were simultaneously detected in different P applications, and 16 were located in 2 or more individual environments.

**Table 4:** **Candidate QTLs and their interactions with environment for yield and P use efficiency traits determined by multi-environment combined analysis**

**Trait**	**QTL**	**Chr** ^**a**^	**Interval** ^**b**^	**Position** **(cM)** ^**c**^	**a** ^**d**^	***h*** ^**2**^ **(a)%** ^**e**^	**ae** ^**f**^	***h*** ^**2**^ **(ae)%** ^**g**^	**Individual environment** ^**h**^
BIOM	*qBIOM1*	1	BIN31-BIN32	26.1	−47.65**	8.6			e1,e3
	*qBIOM2*	2	BIN244-BIN245	32.4	−21.46**	1.7	−19.55* (ae4)	0.5	
	*qBIOM7*	7	BIN1008-BIN1009	59.2	45.50**	7.8	17.35* (ae4)	0.4	e2,e4
	*qBIOM10*	10	BIN1342-BIN1343	34.0	56.95**	12.3			e1,e3
	*qBIOM11*	11	BIN1497-BIN1498	92.4	31.02**	3.6			e1,e2
HI	*qHI1*	1	BIN59-BIN60	62.9	1.85**	9.7			e4
	*qHI2*	2	BIN302-BIN303	79.6	−1.08**	3.4			
	*qHI11*	11	BIN1398-BIN1399	5.9	1.60**	7.4	0.93* (ae4)	0.8	e4
Yield	*qYield2*	2	BIN302-BIN303	79.6	−32.75**	8.1			e1,e2,e4
	*qYield11*	11	BIN1401-BIN1402	15.2	20.18**	3.1			
PUP	*qPUP1*	1	BIN46-BIN47	36.1	−0.10**	4.7			e1,e3
	*qPUP7*	7	BIN1007-BIN1008	55.0	0.09**	3.4			e2
	*qPUP10*	10	BIN1348-BIN1349	37.7	0.11**	5.5			e1,e2,e3
PHI	*qPHI1*	1	BIN59-BIN60	63.4	3.71**	15.8			e2,e4
	*qPHI2*	2	BIN310-BIN311	99.0	−4.18**	20.0			
	*qPHI6*	6	BIN838-BIN839	9.0	−1.49**	2. 6			e1,e3
	*qPHI11*	11	BIN1392-BIN1393	2.2	1.96**	4.4	1.24* (ae4)	0.6	e4
gPUE	*qgPUE4*	4	BIN680-BIN681	106.6	−3.83**	1.4			
strPUE	*qstrPUE1-1*	1	BIN60-BIN61	63.9	24.67**	3.9			
	*qstrPUE1-2*	1	BIN177-BIN178	150.8	27.62**	4.9			e1,e3,e4
	*qstrPUE2*	2	BIN302-BIN303	79.6	−31.30**	6.3			e2,e3,e4
PUEb	*qPUEb2*	2	BIN253-BIN254	39.5	−11.36**	6.4			e3
PUEg	*qPUEg1*	1	BIN143-BIN144	135.9	6.84**	3.6			
	*qPUEg2*	2	BIN302-BIN303	79.6	−13.27**	13.4			e4
	*qPUEg6*	6	BIN946-BIN947	112.7	−7.37**	4.1			
	*qPUEg11*	11	BIN1395-BIN1396	5.2	7.62**	4.4	−4.82* (ae2)	0.6	e2,e4
	*qPUEg12*	12	BIN1612-BIN1613	100.1	9.63**	7.1	−5.14* (ae2)	0.7	e2
PT	*qPT2*	2	BIN294-BIN295	75.7	−0.09**	4.6			e4
	*qPT5*	5	BIN709-BIN710	13.9	−0.09**	4.9			e1,e2,e4
	*qPT8*	8	BIN1130-BIN1131	56.3	−0.09**	5.0			e2,e4
PTE	*qPTE1-1*	1	BIN33-BIN34	30.1	3.26**	6.4			e4
	*qPTE1-2*	1	BIN160-BIN161	140.7	2.59**	4.0			e4
	*qPTE2*	2	BIN310-BIN311	88.0	−4.04**	9.9	−1.92* (ae4)	0.7	
	*qPTE5*	5	BIN708-BIN709	13.4	−2.71**	4.4			e1,e2
	*qPTE8*	8	BIN1131-BIN1132	56.5	−3.45**	7.2			
	*qPTE12*	12	BIN1618-BIN1619	109.3	1.91**	2.2			e2,e4

See Table 1 for abbreviations.

^a^Chromosome the QTL is located on.

^b^The underlined marker is closer to the QTL.

^c^Position (cM) denotes the genetic distance in centiMorgan between the QTL and the first marker on the relevant chromosome.

^d^Additive effect, a negative value indicates that the Zhenshan 97 allele increases phenotypic score.

^e^Phenotypic variation explained by an additive effect.

^f^Additive by environment interaction effect, e1 and e2 represent low P in 2008 and 2009, e3 and e4 represent normal P in 2008 and 2009, respectively.

^g^Phenotypic variation explained by an additive by environment interaction.

^h^The individual environment in which a QTL for the identical trait was detected by individual environment analysis and located in the same or neighboring region listed in the fourth column.

* and **Indicate significance at *P* = 0.05 and *P* = 0.01, respectively.

#### QTLs for BIOM

**Figure 2:**
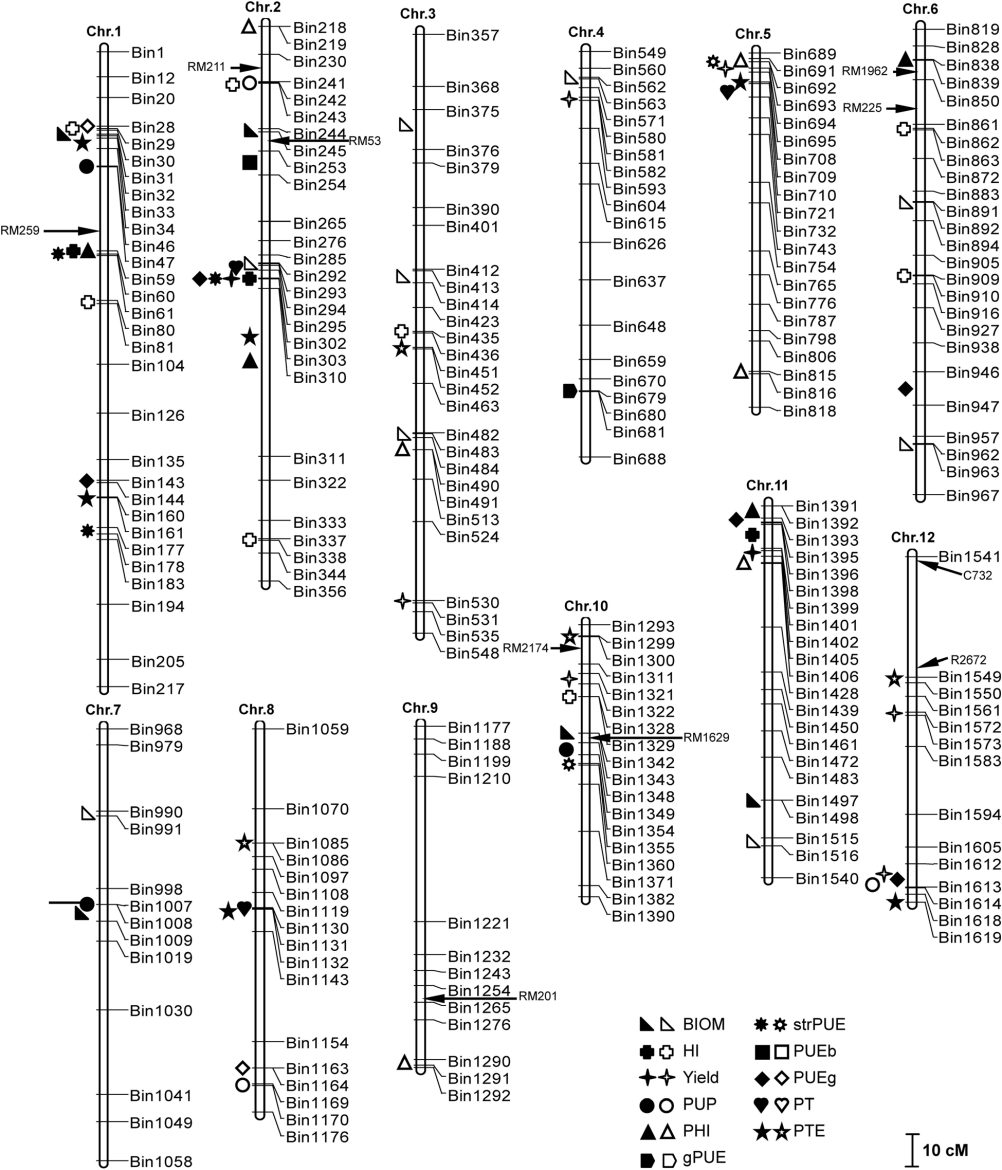
**A genetic linkage map of rice showing the mapping of QTLs with additive effects and epistatic effects.** The sequent SNP markers have been sparsed according to the mapping results. The filled symbols represent the QTLs with additive effects; the open symbols represent the non-QTL locations involved in epistatic interactions.  indicate the QTLs or location detected for BIOM;  for HI;  for Yield;  for PUP;  for PHI;  for gPUE;  for strPUE;  for PUEb;  for PUEg;  for PT; and  for PTE. Markers with arrows indicate a QTL located in a similar region according to RFLP/SSR maps and physical positions in previous studies. Marker RM259 on chromosome 1 [8], RM211 and RM53 on chromosome 2 [47], R1962 and RM225 on chromosome 6 [3,47], RM201 on chromosome 9 [50], R2174 and R1629 on chromosome 10 [3], and C732 and R2672 on chromosome 12 [3,51].

Five QTLs for BIOM were detected, and they explained 34.0% of the total phenotypic variation (Table 4 and Figure 2). Minghui 63, the parent with a high BIOM value, contributed alleles at three QTLs (*qBIOM7*, *qBIOM10*, and *qBIOM11*), and Zhenshan 97 provided two alleles at the other two QTLs. Two QTLs (*qBIOM2* and *qBIOM7*) exhibited interactions with the e4 environmental factor (normal P in 2009), and each interaction explained 0.5% and 0.4% of the total variation, respectively. Except for *qBIOM2*, the remaining four QTLs were detected in two environments across two years.

#### QTLs for HI

Three QTLs for HI were mapped and collectively explained 20.5% of the total phenotypic variation. Minghui 63, with a lower HI relative to Zhenshan 97, provided the two alleles at *qHI1* and *qHI11*, which explained 17.1% of the total variation. The QTL *qHI11* was also detected in the environment e4, and it had a significant interaction with the environment.

#### QTLs for Yield

Two QTLs were identified for Yield, and they jointly explained 11.2% of the total phenotypic variation. The QTL *qYield2* with the low-score parent Zhenshan 97 allele had a large effect and contributed 8.1% of the total variation. The other QTL, which had the Minghui 63 allele, contributed 3.1%. The QTL *qYield2* was mapped in three individual environments. The QTL *qYield2* were detected in three environments across two years.

#### QTLs for PUP

Three QTLs for PUP were identified and explained 13.6% of the total phenotypic variation. Among these three QTLs, the two alleles at *qPUP7* and *qPUP10* were from Minghui 63, which had a higher PUP than Zhenshan 97. The QTLs *qPUP1* and *qPUP10* were identified in multiple environments.

#### QTLs for PHI

Four additive QTLs for PHI were found, and they collectively accounted for 42.8% of the total phenotypic variation. The contribution of each QTL ranged from 2.6% to 20.0%. Minghui 63 provided the alleles at two QTLs, *qPHI1* and *qPHI11*. The alleles for increasing PHI at the other two QTLs, *qPHI2* and *qPHI6*, were from Zhenshan 97. The *qPHI1* with the Minghui 63 allele explained 15.8% of the total variation, whereas the *qPHI2* with the Zhenshan 97 allele contributed 20.0%. A significant interaction was detected only between *qPHI11* and e4. The two QTLs on chromosomes 1 and 6 were found in two environments simultaneously.

#### QTL for gPUE

Only one QTL, *qgPUE4*, was identified for gPUE. It was located in the region BIN680–BIN681 on chromosome 4 and explained 1.4% of the total phenotypic variation.

#### QTLs for strPUE

For strPUE, three QTLs were verified on chromosomes 1 and 2, and together they accounted for 15.1% of the phenotypic variation. The Minghui alleles at two QTLs (*qstrPUE1-1* and *qstrPUE1-2*) and the Zhenshan97 allele at *qstrPUE2* increased strPUE. The two QTLs (*qstrPUE1-2* and *qstrPUE2)* were detected in three environments across two years.

#### QTL for PUEb

Only one QTL (*qPUEb2*) controlling PUEb on chromosome 2 was detected, and it explained 6.4% of the phenotypic variation. This QTL was only found in a single environment.

#### QTLs for PUEg

Five QTLs were detected for PUEg, collectively accounting for 32.6% of the phenotypic variation. The QTL (*qPUEg2*) on chromosome 2 had large additive effect, accounting for 13.4% of the phenotypic variation. The alleles for increasing PUEg came from both Minghui 63 at three QTLs (*qPUEg1*, *qPUEg11*, and *qPUEg12*) and Zhenshan 97 at two QTLs (*qPUEg2* and *qPUEg6*). Two QTLs (*qPUEg11* and *qPUEg12*) had significant interactions with the environment. Three QTLs were located in individual environments simultaneously.

#### QTLs for PT

For PT, three QTLs (*qPT2*, *qPT5*, and *qPT8*) were detected on chromosomes 2, 5, and 8, accounting for 14.5% of the phenotypic variation (Table 4). At these three loci, the alleles from Zhenshan 97 increased the trait. All these QTLs were detected in individual environments.

#### QTLs for PTE

Six QTLs for PTE were detected on chromosomes 1, 2, 5, 8, and 12, and they accounted for 34.1% of the phenotypic variation. The Minghui 63 alleles increased PTE at the three QTLs (*qPTE1-1*, *qPTE1-2*, and *qPTE12*), and the Zhenshan 97 alleles increased the trait score for the remaining QTLs (*qPTE2*, *qPTE5*, and *qPTE8*). The QTL *qPTE2* had a significant interaction with the environment. Among the six QTLs, four were identified in individual environments.

#### Co-location and cluster of QTLs

Thirty-one QTLs were mapped on the same location or clustered in 12 intervals, respectively (Table 5). There were three regions on chromosome 1. The Minghui 63 alleles on two regions (BIN59–BIN61 and BIN143–BIN161) increased the phenotypic score, whereas the region BIN31–BIN47 covered favorable alleles from the two parents. There were three regions on chromosome 2 in which alleles from Zhenshan 97 increased the phenotypic score. The other six regions were located on chromosomes 5, 7, 8, 10, 11, and 12. Among the six regions, Zhenshan 97 contributed alleles for increasing phenotypic score to two regions (BIN708–BIN710 and BIN1130–BIN1132), and Minghui 63 provided alleles in the remaining four regions. Seven of the 12 intervals contained clustered QTLs for yield and PUE traits simultaneously.

**Table 5:** **Chromosomal regions with pleiotropic effects on yield and P use efficiency traits**

**Chromosome**	**Interval**	**Investigated trait** ^**a**^	
		**Yield trait**	**P use efficiency trait**
1	BIN31-BIN47	BIOM(−)	PUP(−), PTE
1	BIN59-BIN61	HI	PHI, strPUE
1	BIN143-BIN161		PUEg, PTE
2	BIN244-BIN254	BIOM(−)	PUEb(−)
2	BIN294-BIN303	HI(−), Yield(−)	strPUE(−), PUEg(−), PT(−)
2	BIN310-BIN311		PHI(−), PTE(−)
5	BIN708-BIN710		PT(−), PTE(−)
7	BIN1007-BIN1009	BIOM	PUP
8	BIN1130-BIN1132		PT(−), PTE(−)
10	BIN1342-BIN1349	BIOM	PUP
11	BIN1392-BIN1402	HI, Yield	PHI, PUEg
12	BIN1612-BIN1619		PUEg, PTE

See Table 1 for abbreviations.

^a^(−) Indicates that Zhenshan 97 allele increases phenotypic score.

#### Detection of epistatic interactions

Twenty-four digenic interactions were detected for eight of the eleven traits (Table 6). Each interaction explained 0.4% to 10.2% of the phenotypic variation. Eighteen of the twenty-four interactions did not involve loci with additive effects. Of the six interactions involving additive QTLs, three occurred between two additive QTLs. The first occurred between the two main QTLs for PUP, *qPUP1* and *qPUP7.* The second additive interaction was between QTLs for PHI and existed between the regions BIN59–BIN60 and BIN1392–BIN1393 for PHI; QTLs for PHI, strPUE, and HI (*qPHI1*, *qstrPUE1-1*, and *qHI1*, respectively) were located on the former region. The third interaction occurred between BIN160–BIN161 and BIN310–BIN311 for QTLs of PTE; the former region contained a QTL for PTE (*qPTE1-2*), and the latter contained the QTLs *qPTE2* and *qPHI2*. Three significant interactions occurred between e4 and QTLs for BIOM, PHI, and PTE (normal P in 2009), and each aae interaction explained 0.5%, 0.5%, and 0.8% of the phenotypic variation, respectively.

**Table 6:** **Epistasis and epistasis by environment interaction on yield and P use efficiency traits**

**Trait**	**QTL**	**Chr**	**Interval**	**Position** ^**a**^	**QTL**	**Chr**	**Interval**	**Position** ^**a**^	**aa** ^**b**^	***h*** ^**2**^ **(aa)%** ^**c**^	**aae** ^**d**^	***h*** ^**2**^ **(aae)%** ^**e**^
BIOM		2	BIN292-BIN293	74.8		6	BIN962-BIN963	128.4	−51.91**	10.2		
		3	BIN375-BIN376	28.2		6	BIN891-BIN892	52.2	−26.30**	2.6		
		3	BIN413-BIN414	76.2		4	BIN562-BIN563	8.2	−29.30**	3.2		
		3	BIN413-BIN414	76.2		3	BIN483-BIN484	125.3	−26.12**	2.6		
		7	BIN990-BIN991	26.4		11	BIN1515-BIN1516	104.2	29.65**	3.3	19.36* (aae4)	0.5
HI	*qBIOM1*	1	BIN30-BIN31	24.9		6	BIN862-BIN863	29.6	−1.23**	4.3		
		1	BIN80-BIN81	78.6		3	BIN435-BIN436	92.3	1.24**	4.4		
		2	BIN242-BIN243	17.7		2	BIN337-BIN338	162.0	−1.81**	9.3		
		6	BIN909-BIN910	75.3		10	BIN1328-BIN1329	22.4	0.99**	2.8		
Yield		3	BIN530-BIN531	177.2		4	BIN580-BIN581	14.7	−26.25**	5.2		
		5	BIN694-BIN695	5.3		10	BIN1321-BIN1322	16.8	−21.15**	3.4		
		12	BIN1572-BIN1573	49.2	*qPUEg12*	12	BIN1612-BIN1613	99.1	−23.47**	4.2		
PUP	*qPUP1*	1	BIN46-BIN47	36.1	*qPUP7*	7	BIN1007-BIN1008	55.0	0.07**	2.3		
		2	BIN241-BIN242	17.5		8	BIN1169-BIN1170	111.4	0.11**	5.8		
	*qPUP10*	10	BIN1348-BIN1349	37.7		12	BIN1613-BIN1614	104.3	0.04*	0.9		
PHI	*qPHI1,qHI1,qstrPUE1-1*	1	BIN59-BIN60	63.4	*qPHI11*	11	BIN1392-BIN1393	2.2	0.58	0.4	1.19* (aae4)	0.5
		2	BIN218-BIN219	0		5	BIN815-BIN816	113.3	−1.65**	3.1		
		3	BIN490-BIN491	129.7		9	BIN1290-BIN1291	104.7	2.18**	5.5		
		5	BIN691-BIN692	3.0		11	BIN1405-BIN1406	17.9	−1.49**	2.6		
strPUE		5	BIN692-BIN693	4.3		10	BIN1354-BIN1355	43.5	−25.93**	4.3		
PUEg		1	BIN28-BIN29	23.9		8	BIN1163-BIN1164	106.4	−8.42**	5.4		
PTE	*qPTE1-2*	1	BIN160-BIN161	140.7	*qPTE2, qPHI2*	2	BIN310-BIN311	88.0	0.84	0.42	2.00* (aae4)	0.8
		3	BIN451-BIN452	97.4		8	BIN1085-BIN1086	35.8	−2.34**	3.30		
		10	BIN1299-BIN1300	3.4		12	BIN1549-BIN1550	38.5	2.49**	3.72		

^a^Position (cM) denotes the genetic distance in centiMorgan between the QTL and the first marker on the relevant chromosome.

^b^Additive by additive effect, a negative value means that recombinant alleles from the two parents increase the phenotypic score, a positive value means that two alleles from an identical parent increase the phenotypic score.

^c^Phenotypic variation explained by an epistatic effect.

^d^Epistasis by environment interaction, e1 and e2 represent low P in 2008 and 2009, e3 and e4 represent normal P in 2008 and 2009, respectively.

^e^Phenotypic variation explained by an epistasis by environment interaction.

* and **Indicate significance at *P* = 0.05 and *P* = 0.01, respectively.

## Discussion

### Interrelationships among PUE traits

Two PUE traits (gPUE and strPUE) may be considered as components of P use efficiency [32]. There were positive correlations among PUEb, PUEg, and gPUE, and among PUEb, PUEg, and strPUE (Table 3 and Figure 1). However, we did not find any regions simultaneously containing QTLs for PUEb, PUEg, and gPUE, or PUEb, PUEg, and strPUE. Moreover, we did not find any co-locations of QTLs for PUEg and gPUE or for PUEb and strPUE. Therefore, P use efficiencies based on the P stored in a specific part of plant (such as gPUE and strPUE) is distinct from those based on the total P in an entire plant (such as PUEg and PUEb). No correlation occurred between strPUE and gPUE, and the co-location of QTLs for the two traits was not observed either. This suggests that strPUE and gPUE are independent from each other. Therefore, our results showed that it might be feasible to improve gPUE and strPUE independently. The majority of accumulated P was distributed in rice grains, and improving gPUE (such as decreasing P concentration in grains) may therefore be a suitable option for reducing plant P demand.

A genotype with tolerance to P deficiency is desirable, with simultaneously high PUP and high PUE [1,3]. Negative correlations between PUP and PUEb, PUEg, gPUE, and strPUE were observed (Table 3, Figure 1). Similar correlations have been previously reported in rice [3] and wheat [15,18,33]. Manske et al. [40] showed that wheat PUEg was negatively correlated with pre-anthesis P accumulation, but positively correlated with post-anthesis P accumulation. In our study, PUEg was negatively correlated with pre-anthesis P accumulation under low P conditions (*r* = −0.27** in 2008, −0.29** in 2009, ** indicate significance at *P* < 0.01) and not correlated with post-anthesis P accumulation under normal P condition. However, gPUE was negatively correlated with post-anthesis P accumulation (*r* = −0.41** under low P in 2008, −0.32** under low P and −0.38** under normal P in 2009). Therefore, the contributions of P accumulations to PUE appear to be different at various growth stages, depending on crop and P level.

The QTLs for PUP did not share any regions with those for the four PUE traits based on grain yield and biomass (PUEb, PUEg, gPUE, and strPUE). Genetic association (close linkage and pleiotropy) and environmental effects are the main causes for the correlations between the traits [41]. Thus, the relationships between PUP and the four PUE traits may not be directly due to genetic association. In fact, P accumulation is involved in root characters, P absorption, and translocation and distributions among various parts of a plant, whereas PUE is associated with biomass production and yield formation, as well as with responses of a plant to P supply (Figure 1).

### Relationships among yield and PUE traits

Seven regions simultaneously controlling yield and PUE traits (Table 5) were identified. These co-locations implied the close association between yield and PUE traits, which is also shown by the correlation analysis (Table 3, Figure 1). Generally, P accumulation and PUEg increased with yield and biomass. As expected, PUP, PUEg, and PUEb were individually positively correlated with yield. Regardless of these correlations, this study did not identify any common regions simultaneously shared by QTLs for PUP, PUEg/PUEb, and Yield. The QTLs for Yield and PUEg shared the interval BIN302–BIN303. This is consistent with the fact that high grain yield often results in high PUEg when P content is stable. We found co-locations for PUEg and PTE and for PUEg and PT, suggesting close relationships of PUEg with PTE and PT. The translocation of P from stems to grains is associated with grain P accumulation and PHI during grain filling [13]. Although Ogawa et al. [42] reported that carbohydrate accumulation is not directly associated with P accumulation in rice grains, increased P translocation to younger panicles may facilitate the grain filling and lead to high grain yield, which is advantageous to PUEg.

In this study, PHI was associated with HI and P contents in grains and straw. The region BIN1392–BIN1402 on chromosome 11 contained four QTLs affecting PHI, PUEg, HI, and Yield. This co-location was consistent with the correlations found in this study and in previous studies [14,33]. In contrast, two regions (BIN59–BIN60 and BIN1392–BIN1399) contained QTLs for HI and PHI (Table 5 and Figure 2). However, the QTLs for PHI were different than those for HI, which suggests a relative independence of the two traits.

Grain yield was taken into account when HI and PUEg were calculated. Higher grain yield may indicate both higher HI and higher PUEg when the P accumulation in an entire plant is the same, as supported by the high correlation between the two traits (Table 3). The two regions on chromosomes 2 and 11 were simultaneously shared by QTLs for HI and PUEg (Table 5). Both the co-location of these QTLs and the correlation between HI and PUEg suggest that PUEg depends on HI to some degree. However, the two traits had specific QTLs that were not common, implying different genetic controls. From the viewpoint of crop physiology, HI is positively associated with grain yield and total biomass, whereas PUEg depends on grain yield, in addition to the P content of an entire plant (Table 3, Figure 1).

Plant P accumulation increases biomass production to a certain extent and *vice versa* [3,15,18], as confirmed by the correlations found in this study (Table 3). Three regions were concurrently shared by QTLs for BIOM and PUP (Table 5). Notably, at least two QTLs for BIOM were not shared by PUP, and no correlation was observed between growth duration and PUP (data not shown), suggesting that the processes for biomass production and P accumulation in rice are not the same. With respect to physiology, biomass production is related to photosynthetic capacity and respiration, and P accumulation is often affected by P absorption and translocation. Moreover, two QTLs for PUEb and BIOM were tightly linked in the region BIN244–BIN254 on chromosome 2, and two regions (BIN294–BIN303 and BIN1392–BIN1402) simultaneously affected PUEg and Yield (Table 5 and Figure 2). This suggests that Yield and PUEg (and BIOM and PUEb) could be concurrently improved by exploring these regions.

### Epistatic and environmental effects for PUE traits

Epistasis is the interaction of genes at different loci, and it plays an important role in the formation of complex traits [43,44]. A dozen pairs of epistatic interactions were detected for PUE traits in this study. Taking PUP as an example, three QTLs explained 13.6% of the phenotypic variation, and three pairs of interactions explained 9.0% of the variation (Tables 4 and 6). This implies that both additive QTLs and epistatic interactions could make substantial contributions to PUE traits. A relatively large proportion of QTLs (33%, 12 of 36) had both individual effects and epistatic interactions (Tables 4 and 6), indicating that interaction is common for yield and PUE traits. Hu et al. [45] and Li et al. [8] have reported similar epistatic interactions.

The development of complex traits integrates genetic and environmental effects. Seven of the 36 additive QTLs (Table 4) and the three epistatic interactions (Table 6) identified in this study interacted significantly with the environment. The seven QTLs with ae effects exhibited various responses to the environment. Several interactions of QTLs with the environment and epistasis with the environment were up-regulated by the particular environment. For example, three additive QTLs (*qBIOM7*, *qHI11*, and *qPHI11*, Table 4) and three pairs of epistatic interactions (Table 6) displayed positive ae and aae effects with the environment e4 (normal P in 2009), suggesting that the QTLs and epistatic interactions were significantly strengthened by the application of P fertilizer.

In contrast, several interactions were down-regulated by the environment. For example, two QTLs controlling PUEg (*qPUEg11*, *qPUEg12)* were down-regulated by e2 (low P in 2009), and two QTLs (*qBIOM2* and *qPTE2*) were down-regulated by e4 (normal P in 2009). The down-regulations of *qPUEg11* and *qPUEg12* by low P and the down-regulation of *qBIOM2* by the normal P application appeared in contrast to the expected behavior, as low P application often results in high PUEg and low BIOM (Table 1). However, for *qPTE2*, the down-regulation by the normal P application was consistent with the reduction of PTE under normal P. Therefore, our results suggest that both QTL and epistatic interactions are differentially expressed in response to the environment.

### Comparisons of QTLs across genetic backgrounds

In previous studies, several populations have been used to detect QTLs associated with PUE in rice, with more attention focused on P deficiency tolerance and relative agronomic traits [3,8,17,45,46]. According to the physical positions in the SNP (Figure 2) and RFLP/SSR maps [28], the QTL *qPUP10* for PUP may share a similar region with a QTL for PUP on chromosome 10 found by Wissuwa et al. [3], which was flanked by the marker R1629. Similarly, the QTL *qPUP1* (BIN46–BIN47) was very close to the marker RM259 on chromosome 1 for root traits, which was found by Li et al. [8]. A QTL interaction linked to PUP on chromosome 2 was located on an interval (BIN241–BIN242) that was tightly linked to marker RM211, which was close to a QTL for root number [47]. These results were consistent with previous studies showing that root traits play an important role in P uptake and tolerance to P deficiency [48,49].

Further increases in grain yield under P deficiency would most likely result from increases in PUP and PHI [33]. The interval BIN1290–BIN1291 on chromosome 9, which was involved in an interaction for PHI, had a similar location to RM201; moreover, Mao et al. [50] identified a QTL for 1000-grain weight on the similar interval (Figure 2). The QTL, *qPHI6* for PHI seems to share a similar region with the QTLs near R1962 on chromosome 6 for tiller number and PUP under P deficiency detected by Wissuwa et al. [3]. This suggests that the QTLs and the interval may be involved in partitioning more P to grains under P deficient conditions.

The interval BIN1549–BIN1550, which was involved in an epistatic interaction for PTE on chromosome 12, was tightly linked to R2672, which was close to two QTLs for P-deficiency tolerance [3] and for amount of stem non-structural carbohydrates per spikelet at heading [51]. Thus, considering the traits linked to and controlled by the same interval, the epistatic effect between BIN1299–BIN1300 and BIN1549–BIN1550 may contribute to the relocations of P and carbohydrates from stems to grains during grain filling.

## Conclusions

In this study, most of the QTLs for the eight PUE traits were different from those for yield traits, indicating different genetic mechanisms underlying high PUE and high grain yield. However, seven regions were shared by yield and PUE traits; therefore, grain yield and PUE traits may be simultaneously improved.

Four QTLs (*qPHI1* and *qPHI2* for PHI, *qPUEg2* for PUEg, and *qPTE8* for PTE) had strong additive effects but no environmental effects (Table 4). Additionally, three QTLs (*qPUP1*, *qPUP10*, and *qPHI6*) were simultaneously found under different genetic backgrounds. These QTLs are promising for improving rice PUE in future breeding research.

This study documented that 12 QTLs and many loci were involved in epistatic interactions, which played substantial roles in determining PUE traits. The observed interactions provide an approach to reveal the genetic networks affecting PUE traits.

## Abbreviations

BIOM: Biomass
gPUE: Grain P use efficiency based on P accumulation in grains
HI: Harvest index
PHI: P harvest index
PT: P translocation
PTE: P translocation efficiency
PUEb: P use efficiency for biomass
PUEg: P use efficiency for grain yield based on P accumulation in the whole plant
PUP: Total P uptake in the whole plant
QTLs: Quantitative trait loci
strPUE: Straw P use efficiency based on P accumulation in straw
